# Melatonin Potentiates Sensitivity to 5-Fluorouracil in Gastric Cancer Cells by Upregulating Autophagy and Downregulating Myosin Light-Chain Kinase

**DOI:** 10.7150/jca.85353

**Published:** 2023-08-21

**Authors:** Xiaorui Shi, Hongxia Li, Zhangyong Dan, Chuanlin Shu, Rumeng Zhu, Qingling Yang, Yi Wang, Huaqing Zhu

**Affiliations:** 1 Laboratory of Molecular Biology, Department of Biochemistry, Anhui Medical University, Hefei 230032, China.; 2 Department of Biological Engineering, School of Life Sciences, Anhui Medical University, Hefei 230032, China.; 3 Department of Oncology, The Third Affiliated Hospital of Anhui Medical University, Hefei 230032, China.; 4 Anhui Province Key Laboratory of Translational Cancer Research, Bengbu Medical College, Anhui 233030, China.

**Keywords:** melatonin, 5-Fluorouracil, autophagy, gastric cancer, MLCK

## Abstract

5-Fluorouracil is an effective chemotherapeutic drug for gastric cancer. However, the acquisition of chemotherapeutic resistance remains a challenge in treatment. Melatonin can enhance the therapeutic effect of 5-fluorouracil; however, the underlying mechanisms are not well understood. We investigated the effects of combinations of melatonin and 5-fluorouracil on the proliferation, migration and invasion of gastric cancer cells. Melatonin significantly potentiated the 5-fluorouracil-mediated inhibition of proliferation, migration and invasion in gastric cancer cells, which potentiates sensitivity to 5-FU by promoting the activation of Beclin-1-dependent autophagy and targeting the myosin light-chain kinase (MLCK) signaling pathway. Previous studies have shown that autophagy might be associated with the MLCK signaling pathway. The autophagy inhibitor, 3-methyladenine, effectively rescued the migratory and invasive capabilities of gastric cancer cells, while also reducing expression level of MLCK and the phosphorylation level of MLC. This indicates that autophagy is involved in tumor metastasis, which may be related to inhibition of the MLCK signaling pathway. Our findings indicate that melatonin can improve the effectiveness of 5-fluorouracil in gastric cancer and could be used as a supplemental agent in the treatment of gastric cancer with 5-fluorouracil.

## Introduction

Gastric cancer (GC) is an aggressive and among the most common malignant tumors worldwide, being the second major cause of cancer-related deaths 1-4. It arises from the inner lining of the stomach and can spread rapidly to the nearby organs and tissues. Although significant progress has been made in the treatment of GC, the mortality rate among patients with the disease remains alarmingly high. The persistently high mortality rate may be attributed to the frequent recurrence of tumors and occurrence of distant metastases 5-9.

5-Fluorouracil (5-FU) is an effective chemotherapeutic drug for GC treatment 10-12. It is a pyrimidine analog and an antimetabolite that inhibits the synthesis of thymine nucleotides, inhibits DNA synthesis, and thereby, the growth of cells, ultimately leading to tumor treatment. However, the development of 5-FU resistance and side effects are two major obstacles to its clinical application 13. Combination of 5-FU with other agents has been used as an effective treatment for GC 14,15.

Melatonin (MLT), also known as pineal hormone, is an indole hormone, mainly produced in the pineal glands of humans and other mammals 16. MLT has anti-inflammatory, antioxidant, free radical-scavenging, antiautophagy and other biological functions 17. Several studies have shown that MLT exhibits chemotherapeutic potential against human cancers by regulating cell growth, proliferation and autophagy 18,19. MLT has been proven to improve drug efficacy, reduce drug toxicity and overcome chemotherapy resistance in cancer; however, the role of MLT in combination with 5-FU in gastric cancer therapy deserves further investigation 20,21.

Myosin light-chain kinase (MLCK) is a key enzyme that regulates a number of cytoskeletal regulatory proteins. It plays a crucial role in regulating the contraction and relaxation of smooth muscle cells by phosphorylating the myosin light chain (MLC) 22. The phosphorylation of MLC triggers a cascade of cytoskeletal regulatory proteins that ultimately lead to changes in the shape and movement of cells. In addition to its role in smooth muscle cells, MLCK is also involved in various cellular processes, such as cell proliferation, migration and invasion, particularly in cancer cells, where it has been shown to promote tumor growth and metastasis. Thus, MLCK is an attractive target for the development of anticancer therapies 23-28.

In this study, we investigated whether MLT can act synergistically with 5-FU in the treatment of GC using in vitro experiments on SGC-7901 and MGC-803 cell lines. We also examined the effects of 5-FU and MLT combination treatment on the expression levels of MLCK and autophagy-related proteins as well as the phosphorylation level of MLC to decipher the mechanisms underlying the effects of these two drugs in SGC-7901 and MGC-803 cells. We show that MLT potentiates the effects of 5-FU in improving gastric cancer sensitivity through multiple signaling pathways, which suggests that this combination therapy may be a more effective method for chemotherapy of GC.

## Materials and Methods

### Cell culture

GC cell lines, SGC-7901 and MGC-803, were obtained from the American Type Culture Collection (ATCC, Manassas, VA, USA). Cells were cultured in Dulbecco's modified Eagle Medium (DMEM; WISENT, Uruguay, South America) containing 10% fetal bovine serum (FBS; WISENT) under 5% CO_2_ at 37 °C. The cells were treated with MLT (1 mM), 5-FU (1 or 10 μg/mL) or their combination for 48 h.

### Cell proliferation assay

Cell proliferation was assayed using the MTT method 29. Hundred microliter cell suspension was added to the wells of 96-well plates at a density of 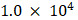
cells per well, with six replicates for each group. Following treatment of cells with MLT (0, 0.125, 0.25, 0.5 or 1 mM) or 5-FU (1, 10, 20, 50 or 100 μg/mL) or with their combination for 24, 48 or 72 h, MTT solution (20 μL; 5 mg/mL) was added to each well. After incubation at 37 °C for 4 h, the MTT solution was replaced with 150 µL dimethyl sulfoxide (DMSO). The optical density (OD) of the solution in each well was measured at 490 nm using a microplate reader after shaking at a low speed for 10 min. The optimum concentrations of MLT and 5-FU were determined based on the MTT assay results.

### Immunofluorescence

GC cells were treated with the drugs and then fixed with 4% paraformaldehyde for 10 min, permeabilized with 0.1% Triton X-100 at 4 °C for 1 min and blocked with 2% bovine serum albumin (BSA) for 1 h at 37 °C. After two washes with phosphate-buffered saline (PBS), the cells were blocked with goat serum at 37 °C for 30 min. The cells were then incubated overnight with the primary antibody Ki67 (Invitrogen, USA) at 4 °C. All samples were incubated with 4′,6-diamidino-2-phenylindole (DAPI) in the dark for 15 min after exposure to goat anti-rabbit IgG (H&L) for 1 h. Finally, the cells were visualized using a fluorescence microscope.

### Western blot analysis

GC cells were lysed in RIPA lysis buffer containing 50 mM Tris (pH 7.4), and the extracted protein was quantified using a BCA Protein Assay Reagent Kit (Beyotime, Shanghai, China). Each sample was then separated by electrophoresis on a 10% SDS-polyacrylamide gel and electroblotted onto a nitrocellulose membrane. The membranes were blocked with 5% skimmed milk in Tris Buffered Saline Tween (TBST) for 2 h and incubated overnight at 4 °C with primary antibodies against MLCK (Santa Cruz Biotechnology, Cat# sc-365352), phosphorylated myosin light chain (pMLC) (Cell Signaling Technology, Cat# 3674), MLC (Cell Signaling Technology, Cat# 8505), p62 (Abmart, Cat# sc-48402), Beclin-1 (Abmart, Cat# T55092), LC3B (Cell Signaling Technology, Cat# 3868) or GAPDH (Santa Cruz Biotechnology, Cat# sc-365062). The PVDF membranes were then washed three times with TBST and incubated with secondary antibodies diluted in 3% milk for 2 h at room temperature. The membranes were washed three times with TBST, and the protein bands were visualized using chemiluminescence.

### Wound-healing assay

A wound-healing assay was performed to detect the migration ability of cells. GC cells were cultured to 100% confluence in 6-well plates. The cell monolayer was scratched with a sterile 100 μL pipette tip, and the wells were washed with phosphate-buffered saline (PBS) to remove the cells dislodged from the wells. The wound gap was observed and photographed using a Leica DM 14000 B microscope. GC cells were treated with 5-FU, MLT or their combination and cultured in a CO_2_ incubator. After 48 h, the culture medium was replaced with PBS, the wound gap was observed and photographed using a microscope equipped with a digital camera, and the width of the wound gap was measured.

### Migration and invasion assay

Migration and invasion abilities of the cells were examined using the Transwell assay performed in Transwell cell culture chambers (8.0 μm pore size) 30,31. Briefly, 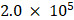 cells were inoculated in a 24-well Transwell chamber for migration experiments or in 10% FBS-containing DMEM in chambers coated with Matrige for invasion experiments. A 200 μL suspension of cells was added at a density of 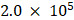 cells/well, and 600 μL of DMEM containing 10% FBS was added to the lower chamber. After the GC cells had adhered to the chamber wall (after 6 h), the complete medium was replaced with serum-free medium, and the cells were treated with 5-FU or a combination of MLT and 5-FU for approximately 40 h. Next, 10% serum (60 μL) was added to the lower chamber and allowed to stand for approximately 8 h. The medium in the upper chamber was washed twice with preheated PBS. The cells were fixed with 4% paraformaldehyde (Biosharp, Beijing, China). Invasive cells on the back of the membrane were stained with 0.1% crystal violet (Biosharp). The cells that could not penetrate the membrane were removed using a wet cotton swab. The numbers of migrating or invading cells were calculated by counting in five random fields of view under a light microscope and expressed as mean ± SE.

### Autophagic flux assay

GC cells were transfected with an mRFP-GFP-LC3 adenovirus (HanBio, Shanghai, China) to detect autophagosomes and autolysosomes. After transfection for 6 h, the cells were treated with 5-FU or a combination of MLT and 5-FU. Autophagic flux was estimated by visualizing the cells using a fluorescence microscope 32,33.

### Statistical analysis

All analyses were performed using the GraphPad Prism 8.0 or ImageJ software. Data are presented as mean ± standard deviation (SD) of triplicate samples or at least three independent experiments. The data were subjected to one-way ANOVA with Tukey's multiple comparison test and unpaired parametric *t*-test and are presented as mean ± SD. Statistical significance was set at *p* < 0.05.

## Results

### Antiproliferative effect of MLT and 5-FU combination on GC cells

**Figure 1:**
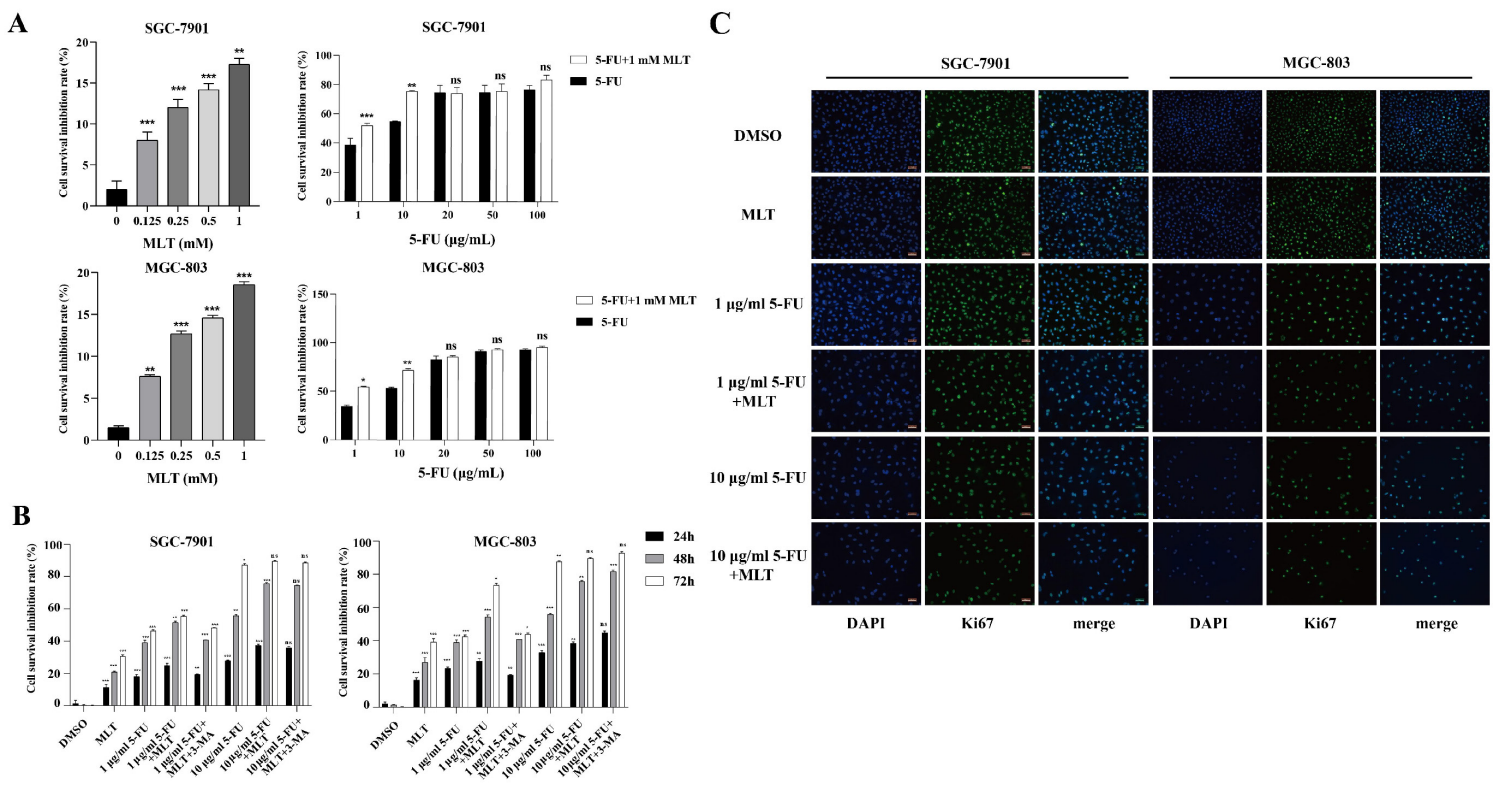
Effect of melatonin (MLT) and 5-fluorouracil (5-FU) combination on the proliferation, migration and invasion of gastric cancer (GC) cells. **(A, B)** The cells were treated with MLT (1 mM) or 5-FU (1 or 10 μg/mL) alone or in combination at specific doses. Following treatment for 24, 48 or 72 h, the rate of inhibition of cell proliferation was determined using the MTT assay. **(C)** Immunofluorescence staining of GCs for Ki67. The experiments were performed three times to ensure accuracy and consistency. One-way ANOVA with Tukey's multiple comparison test was used for statistical analysis. Data are presented as mean ± SD. And statistical significance is indicated using asterisks (*p < 0.05, **p < 0.01, ***p < 0.001).

The rate of inhibition of GC cell proliferation was determined using the MTT assay. SGC-7901 and MGC-803 cells were treated with 0, 0.125, 0.25, 0.5 or 1 mM MLT or 1, 10, 20, 50 and 100 μg/mL 5-FU alone or in combination (1 mM MLT and 1 or 10 μg/mL 5-FU). As shown in Figure 1A, MLT inhibited the proliferation of GC cells in a concentration-dependent manner. However, the proliferation of GC cells was significantly inhibited (38.96%) even when treated with 1 μg/mL 5-FU alone. The inhibition rates were 38.96% with 1 μg/mL 5-FU alone (Figure 1A), 52.68% with a combination of 1 μg/mL 5-FU and 1 mM MLT, and 54.82% with 10 μg/mL 5-FU alone. Thus, for further experiments in this study, we used 1 and 10 μg/mL 5-FU. Moreover, the inhibition rate of treatment with a combination of 10 μg/mL 5-FU and 1 mM MLT in SGC-7901 and MGC-803 cells was >70%. GC cells were treated with MLT (1 mM) or 5-FU (1 or 10 μg/mL) alone, with the MLT and 5-FU combination, or with a combination of 5-FU, MLT and 3-MA for 24, 48 or 72 h (Figure 1B). Compared with the treatment with MLT or 5-FU alone, the combination therapy significantly reduced cell proliferation. The cell survival was inhibited by 85%, which was well over half of the inhibition rate at 72 h. The optimal duration of the drug treatment was 48 h. Therefore, GC cells were treated with 5-FU (1 or 10 μg/mL) alone or in combination with 1 mM MLT for 48 h.

Immunofluorescence staining showed that treatment with the combination of MLT (1 mM) and 5-FU (1 or 10 μg/mL) resulted in fewer Ki-67-positive cells than the treatment with a single drug (Figure 1C) indicating that the combined therapy was more efficient at inhibiting the proliferation of GC cells.

### Effect of treatment with MLT and 5-FU combination on the migration and invasion of GC cells

**Figure 2:**
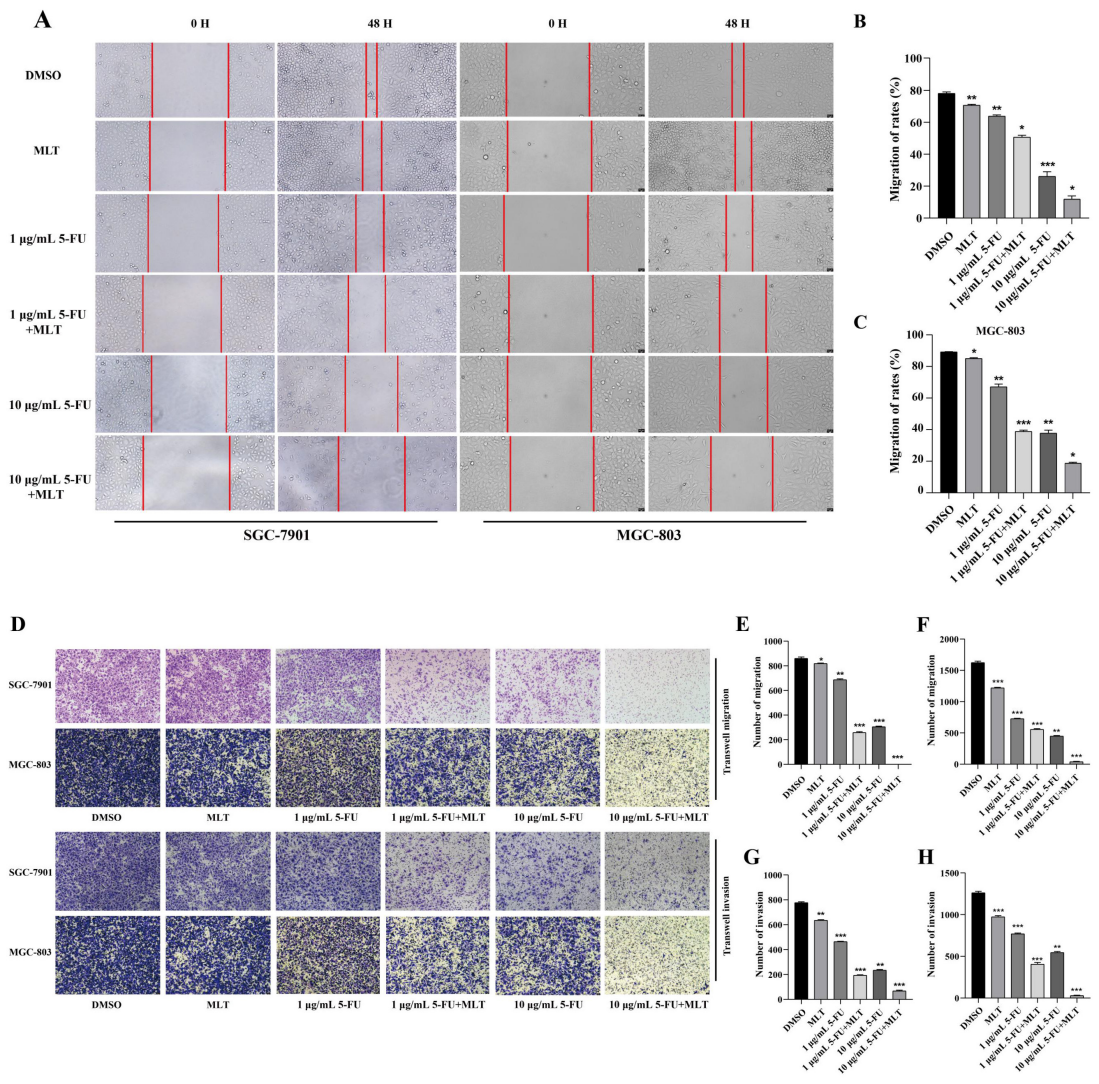
Assessment of cell migration and invasion using wound-healing assay. **(A)** Gastric cancer (GC) cells were cultured to confluence. And the cell monolayer, thus formed, was damaged with a sterile tip of a 100 μL pipette. The cells were further treated with 5-fluorouracil (5-FU; 1 or 10 μg/mL) alone or in combination with melatonin (MLT; 1 mM). **(B, C)** The cells in culture were photographed and the width of the wound was measured at 0 and 48 h after treatment to calculate the percentage of cell migration. **(D)** Results of the Transwell migration experiment. **(E-H)** Invasion of SGC-7901 and MGC-803 cells treated with MLT or 5-FU alone or in combination for 48 h. The cells were photographed. And the percentage of invasive cells was calculated. The experiments were performed three times to ensure accuracy and consistency. One-way ANOVA with Tukey's multiple comparison test was used for statistical analysis. Data are presented as mean ± SD. And statistical significance is indicated using asterisks (*p < 0.05, **p < 0.01, ***p < 0.001).

In addition to investigating the effects of MLT alone on GC cell migration and invasion, we explored the potential synergistic effects of MLT and 5-FU on the migratory and invasion abilities of GC cells using a wound-healing assay. Treatment with MLT (1 mM) or 5-FU (1 or 10 μg/mL) alone inhibited cell migration (Figure 2A), and this effect was enhanced by the combination treatment. After 48 h, the wound in the cell layer tended to close in the DMSO group. However, the gap in the treatments with MLT (1 mM), 5-FU (1 μg/mL) alone or 5-FU (1 μg/mL) in combination with MLT failed to close. Moreover, with an increase in 5-FU concentration, the gap became larger, and the cells were in an unhealthy state. These findings suggested that combination therapy with 1 mM MLT and 1 μg/mL 5-FU significantly inhibited cell migration (Figure 2B, C).

To comprehensively evaluate the effect of the combination treatment on the migration and invasion of GC cells, we performed Transwell migration and invasion assays. These assays enable the evaluation of the ability of cells to migrate through a porous membrane or invade through a matrix-coated membrane, respectively. Migrating and invading cells on the lower surface of the Transwell chambers were stained with crystal violet, and the number of cells was counted under a light microscope. Treatment with MLT (1 mM) alone reduced the number of cells penetrating the membrane compared with the blank control (Figure 2D-H) and treatment with 5-FU (1 or 10 μg/mL) had a more prominent effect. Treatment with an MLT (1 mM) and 5-FU (1 or 10 μg/mL) combination remarkably inhibited GC cells from penetrating Matrigel (Figure 2D-H). Quantitative analysis revealed that the combination treatment significantly enhanced the inhibition of cell migration and invasion compared with the 5-FU alone treatment (Figure 2E-H).

### Effect of treatment with MLT and 5-FU combination on the MLCK signaling pathway in GC cells

**Figure 3:**
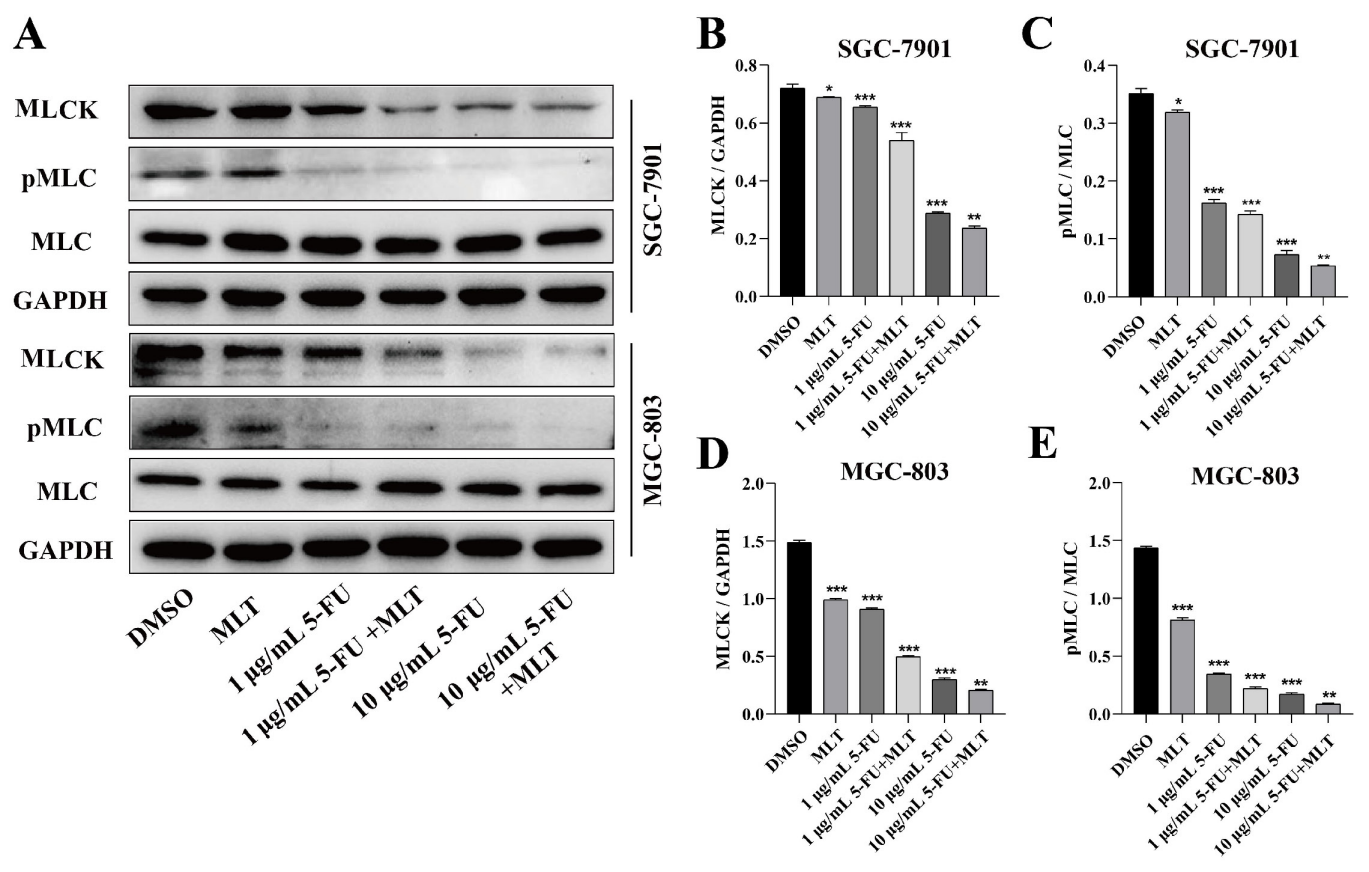
Effect of melatonin (MLT) and 5-fluorouracil (5-FU) on the myosin light-chain kinase (MLCK) signaling pathway in gastric cancer (GC) cells. **(A)** GC cells were cultured and treated with the MLT and 5-FU combination for 48 h. **(B-E)** The expression level of MLCK and the phosphorylation level of MLC were determined using western blotting, which were significantly downregulated by the combination treatment. The experiments were performed three times to ensure accuracy and consistency. One-way ANOVA with Tukey's multiple comparison test was used for statistical analysis. Data are presented as mean ± SD. And statistical significance is indicated using asterisks (*p < 0.05, **p < 0.01, ***p < 0.001).

We investigated the effect of treatment with the MLT and 5-FU combination on the expression level of MLCK and the phosphorylation level of MLC, which is involved in the proliferation, migration and invasion of cancer cells in GC cells using western blotting. The combination treatment markedly suppressed the expression level of MLCK and the phosphorylation level of MLC in GC cells (Figure 3A-E), which indicates that this combination treatment could inhibit the MLCK signaling pathway.

### Effect of treatment with MLT and 5-FU combination on autophagy in GC cells

**Figure 4:**
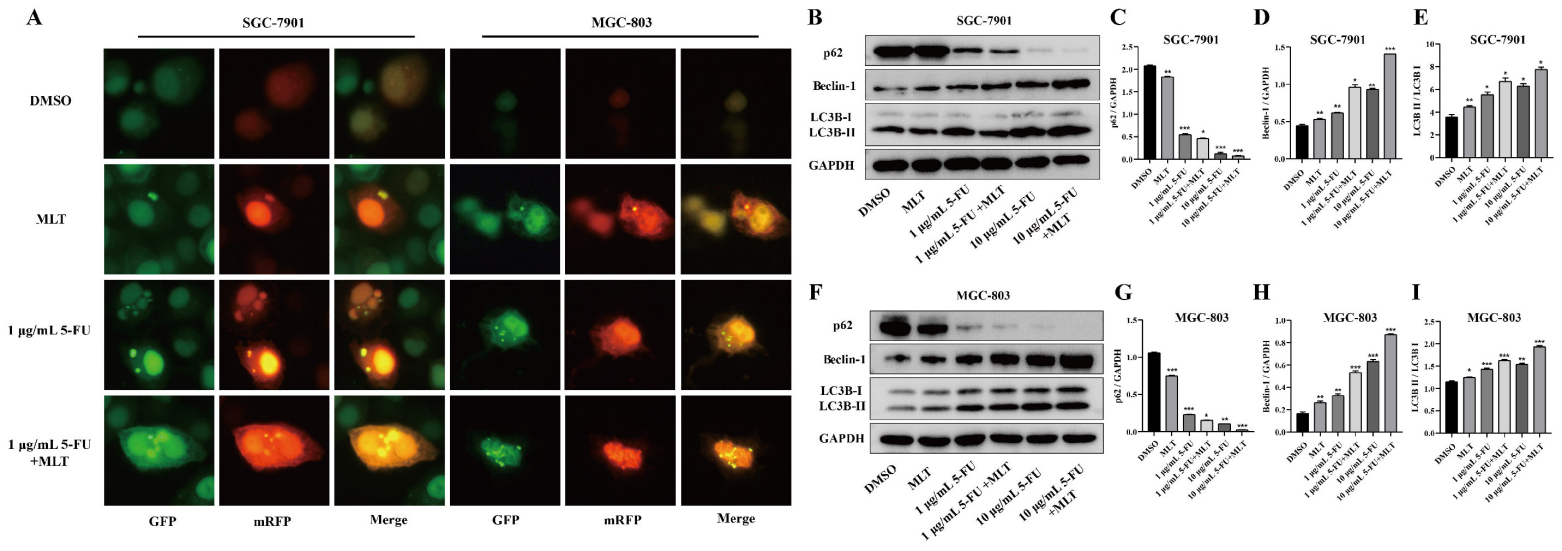
Effect of melatonin (MLT) and 5-fluorouracil (5-FU) combination on autophagy in gastric cancer (GC) cells. **(A)** GC cells overexpressing mRFP-GFP-LC3 were treated with MLT or 5-FU alone or in combination for 48 h. The cells were subsequently observed and photographed using a fluorescence microscope and the picture retains the scale. **(B-I)** In cells subjected to combination treatment, the expression levels of Beclin-1 and LC3B were upregulated and that of p62 was downregulated compared with that in the DMSO group. The experiments were performed three times to ensure accuracy and consistency. One-way ANOVA with Tukey's multiple comparison test was used for statistical analysis. Data are presented as mean ± SD. And statistical significance is indicated using asterisks (*p < 0.05, **p < 0.01, ***p < 0.001).

Autophagy plays an essential role in the progression of GC 34,35. To determine whether the synergistic inhibition of cell growth by 5-FU and MLT was related to the activation of autophagy, we measured autophagy flux. After treatment with MLT (1 mM) or 5-FU (1 or 10 μg/mL) alone or in combination for 48 h, GC cells transfected with MRFP-GFP-LC3 tandem fluorescent protein adenovirus were photographed using a fluorescence microscope (Figure 4A). Compared with the solvent control group, the yellow bright spots were increased in the treatment with MLT or 5-FU alone, and the effect was further increased in the combination treatment. This indicates an increase in autophagy upon treatment with the 1 mM MLT and 1 μg/mL 5-FU combination.

We further investigated the effect of the combination of MLT and 5-FU on the expression levels of autophagy-related proteins. The expression levels of LC3B and Beclin-1 were markedly upregulated, whereas that of p62 was downregulated upon treatment with the MLT (1 mM) and 5-FU (1 μg/mL) combination (Figure 4B-I). This suggests that the MLT and 5-FU combination therapy can promote autophagy in gastric cancer cell lines.

### Effects of treatment with MLT and 5-FU combination in GC cells were alleviated by 3-methyladenine

**Figure 5:**
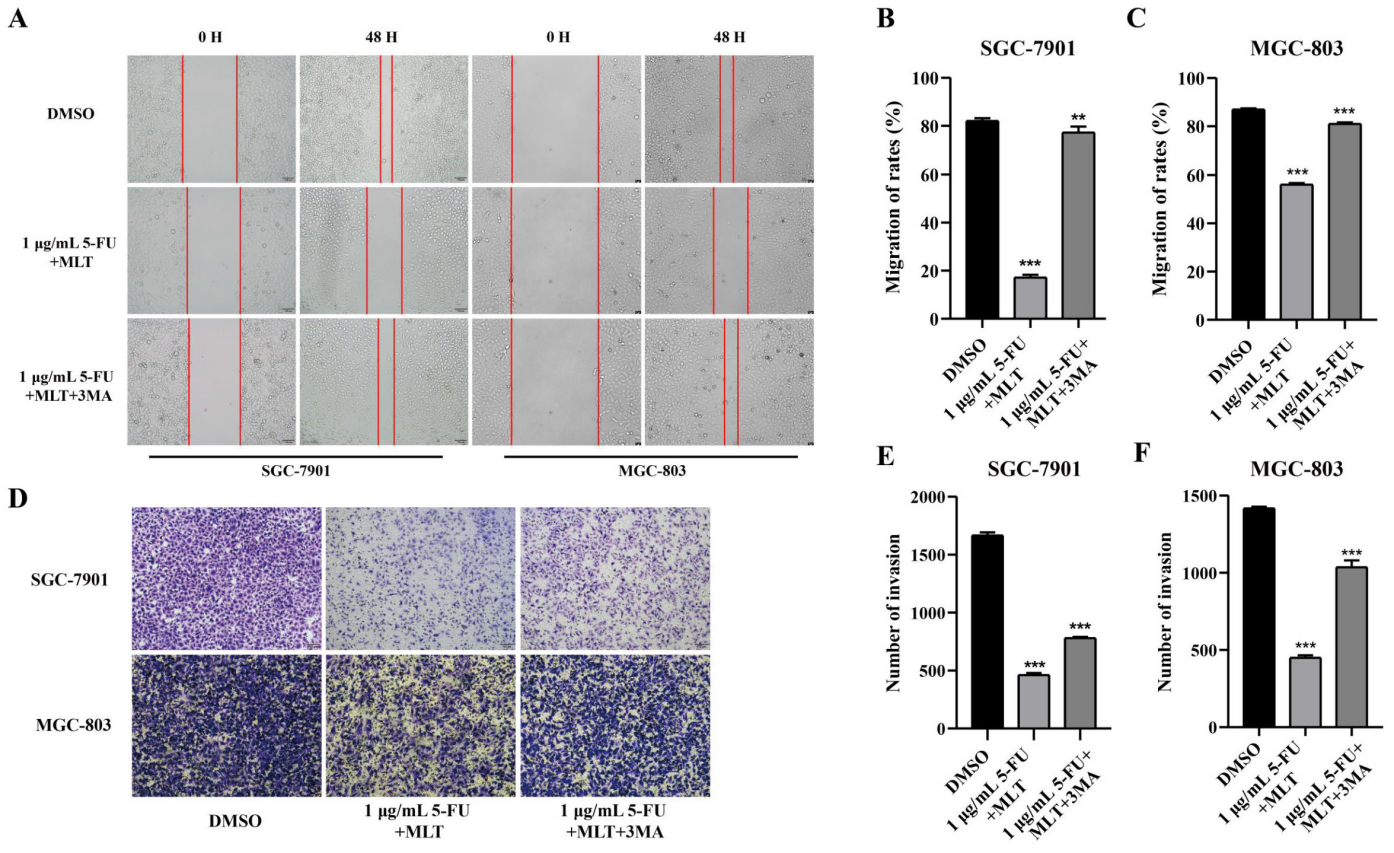
Effect of 3-methyladenine (3-MA) in combination with melatonin (MLT) and 5-fluorouracil (5-FU) on proliferation, migration and invasion of gastric cancer (GC) cells. **(A-C)** A wound-healing assay was performed to assess cell migration. GC cells were cultured to confluence. And the cell monolayer was scratched with a sterile tip of a 100 μL pipette. The cells were then left untreated or were treated with 5-fluorouracil (5-FU) and melatonin (MLT) or 5-FU, MLT and 3-MA combinations. The cells in culture were photographed and the width of the wound was measured after 48 h of treatment. **(D)** Cell invasion was analyzed following treatment with either the 5-FU and MLT combination or the 5-FU, MLT and 3-MA combination for 48 h. The invasive cells were observed and photographed. **(E, F)** Percentage of invasive cells. The experiments were performed three times to ensure accuracy and consistency. One-way ANOVA with Tukey's multiple comparison test was used for statistical analysis. Data are presented as mean ± SD. And statistical significance is indicated using asterisks (**p < 0.01, ***p < 0.001).

Because cell proliferation, migration and invasion are pivotal processes in cancer 36,37, the autophagy inhibitor 3-methyladenine (3-MA) was used as a key factor for cancer regulation. After 48 h of treatment, the wound gap in the solvent control group tended to close, whereas that in the drug treatment group did not (Figure 5A-C). Moreover, compared with the drug treatment group, the autophagy inhibitor 3-MA (2.5 mM) caused the wound gap to close, indicating that 3-MA promoted the migration of GC cells. The Transwell assay was performed to investigate whether 3-MA inhibited the invasive ability of the cells. The number of cells passing through the Transwell chambers was significantly reduced in the MLT and 5-FU combination treatment groups (Figure 5D-F) compared with that in the DMSO control group. This suggests that the combination of MLT and 5-FU had a synergistic inhibitory effect on the migration and invasion of GC cells. Furthermore, treatment with 3-MA resulted in an increase in the number of cells passing through the membrane compared with that in the drug treatment group. This finding suggests that 3-MA promotes the invasion of GC cells.

**Figure 6:**
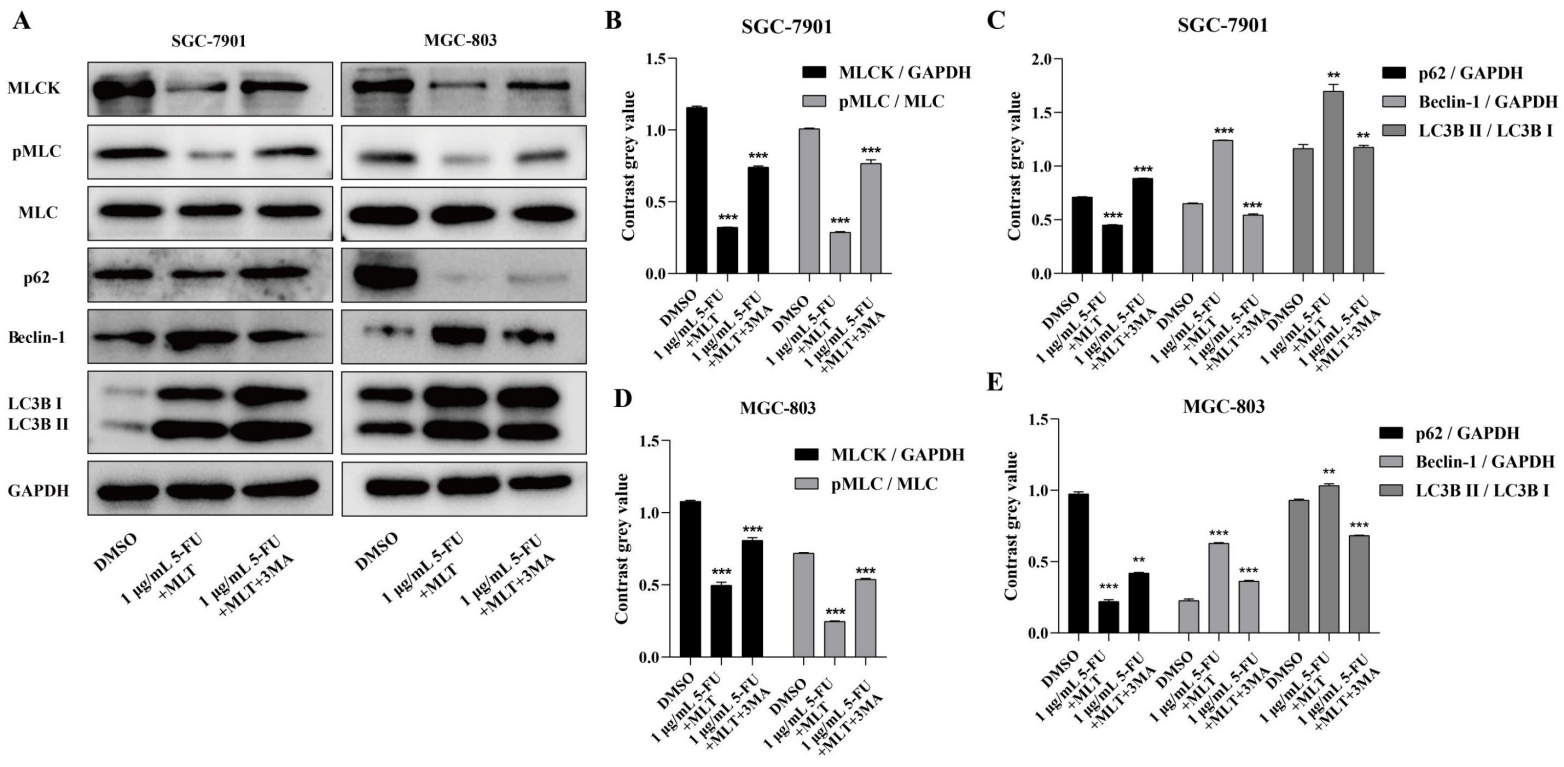
** (A)** Western blot analysis to assess the expression levels of autophagy-related proteins in response to treatment with the autophagy inhibitor, 3-methyladenine (3-MA). **(B-E)** The expression level of MLCK, the phosphorylation level of MLC and the expression level of p62 were significantly downregulated and the expression levels of Beclin-1 and LC3B were significantly upregulated in comparison with the DMSO group; however, these results were reversed when the cells were treated with 2.5 mM MLT. The experiments were performed three times to ensure accuracy and consistency. One-way ANOVA with Tukey's multiple comparison test was used for statistical analysis. Data are presented as mean ± SD. And statistical significance is indicated using asterisks (**p < 0.01, ***p < 0.001).

We also investigated the effects of 3-MA on the expression level of MLCK, the phosphorylation level of MLC and the expression levels of three key autophagy proteins (p62, LC3B and Beclin-1) in GC cells. The expression levels of LC3B and Beclin-1 were upregulated and those of MLCK, MLC phosphorylation and p62 were downregulated upon treatment with the MLT (1 mM) and 5-FU (1 or 10 μg/mL) combination but these effects were reversed when the cells were treated with 2.5 mM 3-MA (Figure 6A-E). These results indicated the role of autophagy in the inhibition of cell proliferation, migration and invasion by MLT and 5-FU combination treatment.

## Discussion

Owing to early metastasis 38-40, patients with GC tend to have a poor prognosis and short lifespan. Metastasis involves the escape and migration of tumor cells and invasion of the basement membrane 41. Understanding the underlying conditions that promote metastasis of GC cells is crucial for developing effective therapeutic options to control the growth and spread of GC.

5-FU is widely recognized as the first-line medicine for chemotherapy of GC owing to its ability to effectively improve overall survival rates. This drug works by inhibiting DNA and RNA synthesis in cancer cells, ultimately leading to their death. However, resistance to 5-FU results in a limited therapeutic effect 42. Thus, there is an urgent need to improve the efficacy of 5-FU. Many studies have shown that MLT has important oncostatic properties in a myriad of cancers, including gastric and liver cancers 43-46. Shen et al. reported that MLT exerts antiproliferative and proautophagic effects on head and neck squamous cell carcinoma (HNSCC) and can be used as a therapeutic agent for HNSCC 47. In this study, we investigated the effects of the MLT and 5-FU combination. This combination increased the rate of survival inhibition and cytotoxic effects in SGC-7901 and MGC-803 GC cells. These results showed that treatment with 5-FU alone inhibited cell migration and invasion, whereas its combination with MLT enhanced the inhibitory effects.

MLCK is the primary protein promoting cell contractility 48, and MLC phosphorylation remarkably improves the migration and invasive abilities of GC cells 49. Leiomyosarcomas, with high proliferative activity, exhibit high MLCK expression 50. Numerous studies have established the pivotal role of MLCK in tumorigenesis, highlighting its potential as a therapeutic target for various cancer types 51. MLCK is encoded by *MYLK*, which controls smooth muscle contraction through MLC phosphorylation. Myosin II-mediated actomyosin contraction is responsible for force production during cell migration and plays a role in membrane protrusion at the frontier 52. Because MLCK is a key enzyme in the phosphorylation of MLC, it is thought to be required for cell migration and invasion. In our study, treatment of GC cells with 5-FU or a combination of MLT and 5-FU for 48 h resulted in the downregulation of MLCK expression. Simultaneously, the expression level of MLCK and the phosphorylation level of MLC were significantly decreased. This indicated that MLCK and MLC phosphorylation markedly improve the migration and invasive abilities of GCs.

Cancer cell migration and invasion are associated with autophagy 3,53. Autophagic flux analysis revealed that treatment with the MLT and 5-FU combination enhanced the cytotoxic activity against GC cells by inducing autophagy. Recent studies have suggested that autophagy also contributes to cancer cell migration and invasion by regulating various signaling pathways and modulating the tumor microenvironment 54,55. Our results showed that the expression levels of LC3B and Beclin-1 were increased, the expression level of p62 was decreased. And cell migration and invasion were hindered in GC cells treated with the 5-FU and MLT combination. Thus, the combination treatment could promote autophagy in SGC-7901 and MGC-803 cells. The autophagy inhibitor, 3-MA, could rescue the migration and invasive abilities of GC cells. The expression level of MLCK and the phosphorylation level of MLC were enhanced in the 3-MA treatment group compared with that in the drug treatment group. The expression levels of Beclin-1 and LC3B were decreased. And those of p62 were increased in the 3-MA treatment group compared with those in the drug treatment group. This indicated that autophagy is involved in tumor metastasis, which may be related to the inhibition of the MLCK signaling pathway.

Our study provides evidence that the combined treatment with 5-FU and MLT can lead to the dysfunction of GC cells, and would provide valuable insights into genetic markers related with GC. It is well known that ncRNAs are indeed closely related to the diagnosis and treatment of tumor. Zhao et al. present a new deep learning algorithm named as graph convolutional network with graph attention network (GCNAT) to predict the potential associations of disease-related metabolites. This kind of deep learning algorithm may help in the diagnosis and treatment of GC 56-61.

## Conclusion

**Figure 7:**
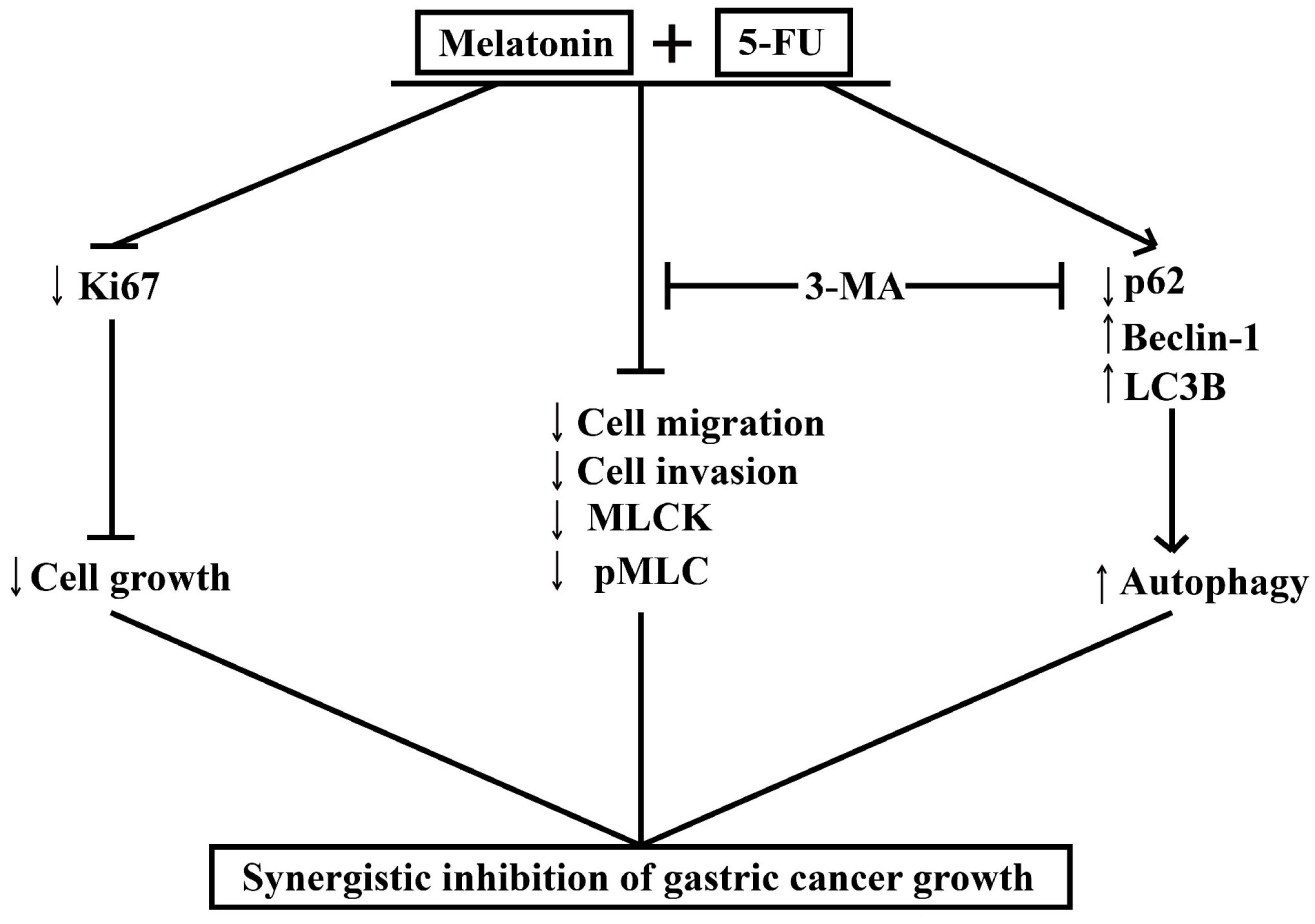
Melatonin synergizes the chemotherapeutic effect of 5-FU to inhibit the growth of gastric cancer by targeting multiple signaling pathways. Melatonin in combination 5-FU suppressed the expression level of Ki67, and inhibited the expression level of MLCK, the phosphorylation level of MLC and the expression level of p62, and promoted the expression levels of Beclin-1 and LC3B, thereby suppressed the MLCK signaling pathway and promoted autophagy signal pathway. And autophagy inhibitors 3-MA could reverse cell migration, cell invasion, the levels of MLCK expression, MLC phosphorylation and autophagy-related proteins expression.

MLT synergizes with 5-FU to inhibit the growth of GC cells by enhancing their antiproliferative, antimigratory, and proautophagic activities. Our study demonstrates that MLT enhances the sensitivity of GC cells to 5-FU by enhancing autophagy and downregulating MLCK. We examined the effects of the combination of MLT and 5-FU on the expression level of MLCK, the phosphorylation level of MLC and the expression levels of autophagy-related protein and in GC cells. The MLT and 5-FU combination reduced cell migration and invasion by enhancing autophagy and downregulating the expression level of MLCK, which was shown to be associated with enhanced autophagic activity. Moreover, the autophagy inhibitor, 3-MA, rescued the expression level of MLCK. Our study provides evidence that the combined treatment with 5-FU and MLT can lead to the dysfunction of GC cells. We showed that 5-FU in combination with MLT reduced autophagy, cell migration and invasion, thereby, minimizing the damage in GC, and that this effect was restrained through the regulation of autophagy and downregulation the expression level of MLCK (Figure 7). These findings provide new insights into the molecular mechanisms by which melatonin synergized GC cells to 5-FU treatment and suggest that such a combinatorial treatment might potentially become a more effective way in GC therapy.

## Supplementary Material

Supplementary figures.
