# Supplementary material for: Melatonin Potentiates Sensitivity to 5-Fluorouracil in Gastric Cancer Cells by Upregulating Autophagy and Downregulating Myosin Light-Chain Kinase
Source: J Cancer. 2023 Aug 21;14(14):2608–18. doi: 10.7150/jca.85353 (PMC10539390; doi:10.7150/jca.85353)
Supplement: Supplementary file 1 — Supplementary figures. [file jcav14p2608s1.pdf]

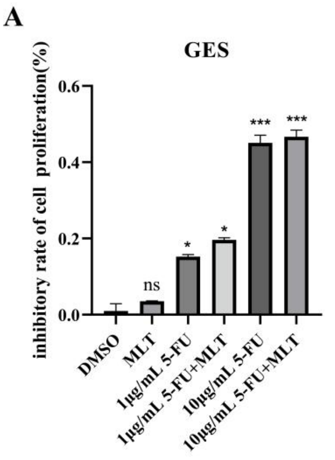

Supplemented Figure 1. A The GES cells were treated with MLT (1 mM) or 5-FU (1 or 10 μg/mL) alone, or in combination at specific doses. And the rate of inhibition of cell proliferation was determined using the MTT assay. The experiments were performed three times to ensure accuracy and consistency. One-way ANOVA with Tukey's multiple comparison test was used for statistical analysis. Data are presented as mean ± SD, and statistical significance is indicated using asterisks (*$*p < 0.05$, $***p < 0.001$*).

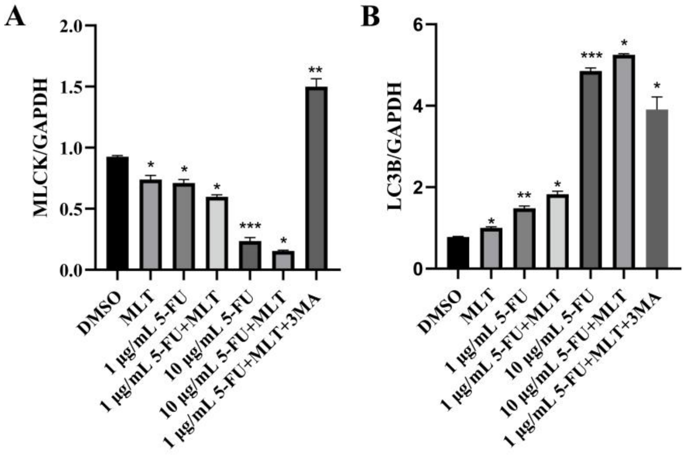

Supplemented Figure 2. Effect of melatonin (MLT) and 5-fluorouracil (5-FU) on the mRNA levels of myosin light-chain kinase (MLCK) and LC3B in gastric cancer (GC) cells. **A,B** The mRNA expression level of MLCK was significantly downregulated, and the mRNA expression level of LC3B was markedly upregulated by the combination treatment. The experiments were performed three times to ensure accuracy and consistency. One-way ANOVA with Tukey's multiple comparison test was used for statistical analysis. Data are presented as mean ± SD, and statistical significance is indicated using asterisks (*$*p < 0.05$, $**p < 0.01$, $***p < 0.001$*).

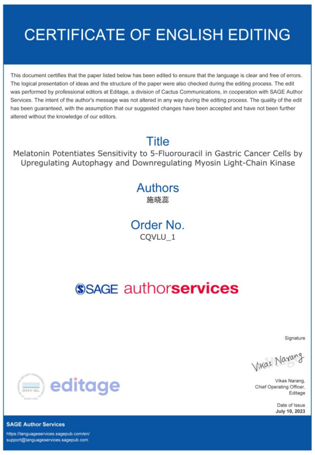

CERTIFICATE OF ENGLISH EDITING

This document certifies that the paper listed below has been edited to ensure that the language is clear and free of errors. The logical presentation of ideas and the structure of the paper were also checked during the editing process. The edit was performed by professional editors at Editage, a division of Cactus Communications, in cooperation with SAGE Author Services. The intent of the author's message was not altered in any way during the editing process. The quality of the edit has been guaranteed, with the assumption that our suggested changes have been accepted and have not been further altered without the knowledge of our editors.

Title
Melatonin Potentiates Sensitivity to 5-Fluorouracil in Gastric Cancer Cells by Upregulating Autophagy and Downregulating Myosin Light-Chain Kinase

Authors
施晓蕊

Order No.
CQVLU_1

SAGE authorservices

Signature

Vikas Narang

Vikas Narang,
Chief Operating Officer,
Editage

editage

Date of Issue
July 10, 2023

SAGE Author Services
https://languageservices.sagepub.com/en/
support@languageservices.sagepub.com

Supplemented Figure 3. Certificate of English editing
